# Mg-incorporated sorbent for efficient removal of trace CO from H_2_ gas

**DOI:** 10.1038/s41467-023-42871-6

**Published:** 2023-11-03

**Authors:** Gina Bang, Seongmin Jin, Hyokyung Kim, Kyung-Min Kim, Chang-Ha Lee

**Affiliations:** 1 Department of Chemical and Biomolecular Engineering, Yonsei University, Seoul, Republic of Korea; 2 Institute of Chemical Sciences and Engineering, École Polytechnique Fédérale de Lausanne (EPFL), Lausanne, Switzerland; 3 Department of Biochemical Engineering, Gangneung-Wonju National University, Gangneung, Republic of Korea

**Keywords:** Chemical engineering, Porous materials, Fuel cells

## Abstract

Removal of trace CO impurities is an essential step in the utilization of Hydrogen as a clean energy source. While various solutions are currently employed to address this challenge, there is an urgent need to improve their efficiency. Here, we show that a bead-structured Mg, Cu, and Ce-based sorbent, Mg_13_CuCeO_*x*_, demonstrates superior removal capacity of trace CO from H_2_ with high stability. The incorporation of Mg boosts sorption performance by enhancing the porous structure and Cu^+^ surface area. Remarkably, compared to existing pelletized sorbents, Mg_13_CuCeO_*x*_ exhibits 15.5 to 50 times greater equilibrium capacity under pressures below 10 Pa CO and 31 times longer breakthrough time in removing 50 ppm CO in H_2_. Energy-efficient oxidative regeneration using air at 120 °C allows its stable sorption performance over 20 cycles. Through in-situ DRIFTS analysis, we elucidate the reaction mechanism that Mg augments the surface OH groups, promoting the formation of bicarbonate and formate species. This study highlights the potential of MgCuCeO_*x*_ sorbents in advancing the hydrogen economy by effectively removing trace CO from H_2_.

## Abstract (web summary)

The challenge of trace CO removal from H_2_ remains at the forefront of the hydrogen economy. Here, the authors demonstrated exceptional CO removal using a highly stable, hierarchically structured sorbent bead, Mg_13_CuCeO*x*. Mechanistic analysis showed the pivotal role of Mg in enhancing CO sorption.

## Introduction

Hydrogen (H_2_) has emerged as a key player in the pursuit of clean energy and decarbonization, especially for industries in which the reduction of greenhouse gases is arduous and alternative solutions are limited. The International Energy Agency (IEA) reported that the annual H_2_ demand reached 94 million metric tons (MtH_2_ yr^−1^) in 2021^1^, representing a more than fivefold increase since 1975. Industries such as heavy transport, shipping, aviation, and heavy industry have become progressively reliant on H_2_^2^, while strategic frameworks developed by the European Commission^3^ and the U.S. Department of Energy (DOE)^4^ have accelerated the adoption of H_2_ as an energy vector in various regions.

To ensure the successful utilization of H_2_, particularly in proton exchange membrane fuel cells (PEMFCs), eliminating trace impurities such as carbon monoxide (CO) is of critical importance^5,6^. Because CO can bind to a platinum catalyst, it significantly reduces the ability of the catalyst to facilitate the target electrochemical reactions^7^, and this deterioration is observed even at very low CO concentrations^8^. Therefore, the International Organization for Standardization (ISO) has introduced the ISO 14687:2019 standard, which limits the CO content in fuel-cell-grade H_2_ to below 0.2 ppm^5^. Numerous H_2_ purification methods have been proposed to comply with these stringent H_2_ purity standards, including membrane separation^5,9^, pressure (vacuum) swing adsorption (P(V)SA)^10,11^, and preferential oxidation of CO (PROX)^12,13^. However, these technologies lead to a loss of H_2_ even when targeting CO concentrations at a more lenient threshold than that imposed by ISO standards. CO can be successfully removed using more complex or combined separator systems, but this requires greater energy consumption and higher capital costs^14^. Moreover, the CO-PROX process, which continuously converts CO into carbon dioxide (CO_2_), has two main concerns: (1) CO_2_ decomposition can occur through in-situ reactions with H_2_ (CO_2_ + H_2_ → H_2_O + CO) or the electro-reduction of CO_2_ (CO_2_ + 2H^+^ + 2e^−^ → CO + H_2_O) in PEMFCs^7^, and (2) it produces CO_2_, meaning that it fails to meet ISO standards requiring CO_2_ levels lower than 2 ppm^15^. Therefore, the CO-PROX process needs reaction energy and an additional separation unit for the removal of CO_2_ and excess O_2_ with additional regeneration energy. As a result, compliance with ISO standards using current H_2_ purification methods is difficult due to the requirement of complex and energy-intensive processes.

To overcome these limitations, it is essential to develop sorbents with a robust CO sorption capacity (i.e., within the ppm range) that do not generate CO_2_ and thus avoid the need for additional separation processes. However, the majority of CO sorbent research to date has concentrated on enhancing CO sorption within a pressure range of 100–200 kPa, which is more useful for CO production than for removal^16,17^. Additionally, H_2_ products from H_2_ production or purification processes contain a few percent or ppm of CO (Supplementary Table 1). Notably, while previous studies on powder sorbents have demonstrated a high CO sorption performance at 100–200 kPa of CO^16^, a research gap exists regarding CO sorption capacity at ppm levels. Simultaneously, investigations into applying them in a stable pellet form for practical process applications are also lacking. The pelletization of powder sorbents often results in reduced sorption capacity and stability due to a decrease in surface area and porosity in pellets^18^. Given these considerations, the development of feasible materials that not only offer high sorption capacity at CO ppm levels but also maintain process stability in pelletized form is necessary for practical applications.

Cu^+^ has been widely employed to facilitate CO sorption through both π-back donation and σ-bond formation^19^, but sorbents containing Cu^+^ experience aggregation during regeneration and oxidation when exposed to air^16,20^. In fact, a material suitable for CO sorption needs a high CO sorption capacity, oxidation resistance, and recycling ability, even at ultra-low CO concentrations. To address these challenges, a range of strategies have been employed to stabilize Cu^+^ in sorbents and catalysts, with one approach involving Cu–Ce systems. These systems exploit the Cu^+^ + Cu^2+^ ↔ Ce^4+^ + Ce^3+^ redox cycle as an oxygen buffer, stabilizing Cu^+^ ions^21^. Cu–Ce systems have exhibited great promise for CO removal applications, in particular as catalysts for low-pressure CO-PROX reactions^22^.

It has been reported that incorporating magnesium into a Cu–Ce system promotes redox mechanisms, thus enhancing the water–gas shift reaction^5^. In particular, previous study has suggested that the introduction of Mg into Cu increases oxygen mobility^23^, which can intensify the interaction between CO and the material surface^24^. Moreover, Mg-based materials with hierarchical structures have recently gained traction in enhancing sorption due to their high porosity^25,26^, while also preventing the aggregation of metal alloys^27^ and improving stability, strength^28^, plasticity, and corrosion resistance^29,30^. Additionally, cyclic sorption stability has been achieved with the fabrication of a hierarchical spherical structure composed of Mg and Ce, further demonstrating the advantages of hierarchical configurations^31^.

In this work, we synthesized a bead-structured Mg, Cu, and Ce-based sorbent (denoted as CuCeO_*x*_ and MgCuCeO_*x*_) that exhibited a significantly enhanced CO sorption performance at low pressure (i.e., <10 Pa). The role of Mg in MgCuCeO_*x*_ was examined by adjusting the Mg content and evaluating the resulting textural properties and morphological features of the sorbent. The physical properties of samples including Brunauer–Emmett–Teller (BET) surface areas were determined by the N_2_ isotherms at 77 K. Since the total Cu surface area could be evaluated by the N_2_O chemisorption, X-ray photoelectron spectroscopy (XPS) and N_2_O chemisorption analysis were utilized to investigate the Cu^+^ surface area (denoted as *S*_Cu+_). CO sorption isotherms at 25 °C and breakthrough experiments using 50 ppm CO were conducted to assess the CO removal performance of MgCuCeO_*x*_, which was compared with CuCeO_*x*_ as a reference. Temperature-programmed desorption (TPD) experiments were carried out under oxygen or inert gas conditions to determine the influence of the regeneration gas, and the cyclic stability and working capacity of the sorbent for reactive CO removal under various regeneration conditions were investigated using thermogravimetric analysis (TGA). The CO sorption mechanisms of MgCuCeO_*x*_ and CuCeO_*x*_ with 50 ppm CO were determined using in-situ diffuse reflectance infrared Fourier transform spectroscopy (DRIFTS), which provided insights into the role of Mg in MgCuCeO_*x*_ and identified the pathway for the surface reaction involving CO.

## Results

### Physicochemical properties of the Mg-promoted CuCeO_*x*_ sorbent

Mg_*α*_CuCeO_*x*_ beads and CuCeO_*x*_ powders were successfully prepared by using a sol-gel combustion-assisted method. (Here, *α* represents the weight percentage of Mg relative to the total metal content.) Table 1 summarizes the textural properties of the CuCeO_*x*_ and MgCuCeO_*x*_ samples, as determined through the N_2_ adsorption/desorption isotherms at 77 K depicted in Supplementary Fig. 2a. The isotherm curve for CuCeO_*x*_ was classified as Type II using the BET isotherm classification, indicating a nonporous or macroporous material^32^. On the other hand, the isotherm curves for the MgCuCeO_*x*_ samples were classified as Type IV with an H3 hysteresis loop, which is typical of a mesoporous material^33^. In Supplementary Fig. 2b, the pore size distribution determined using the Barrett–Joyner–Halenda (BJH) method revealed the development of mesopores with the addition of Mg. Notably, Mg_13_CuCeO_*x*_ had a narrow pore distribution centered around a diameter of approximately 2 nm. Table 1 shows that mesopores were more common than micropores in all of the samples. In addition, the surface area (52–232 m^2^ g^−1^), micropore volume (0.012–0.075 cm^3^ g^−1^), and mesopore volume (0.086–0.389 cm^3^ g^−1^) of the samples increased noticeably with higher levels of Mg. Thus, the addition of Mg led to the development of a Cu-Ce structure with numerous pores and a high surface area.

**Table 1:** Textural properties of the as-prepared sorbent samples

Sorbents	BET surface area (m^2^ g^−1^)	Average pore diameter (nm)	Micropore volume^a^ (cm^3^ g^−1^)	Mesopore volume^b^ (cm^3^ g^−1^)
CuCeO_*x*_	52	3.8	0.012	0.086
Mg_13_CuCeO_*x*_	102	2.9	0.029	0.149
Mg_23_CuCeO_*x*_	117	3.6	0.035	0.233
Mg_52_CuCeO_*x*_	232	3.4	0.075	0.389

^a^by D-R method from 77 K N_2_ isotherm.

^b^by BJH method from 77 K N_2_ isotherm.

**Fig. 1:**
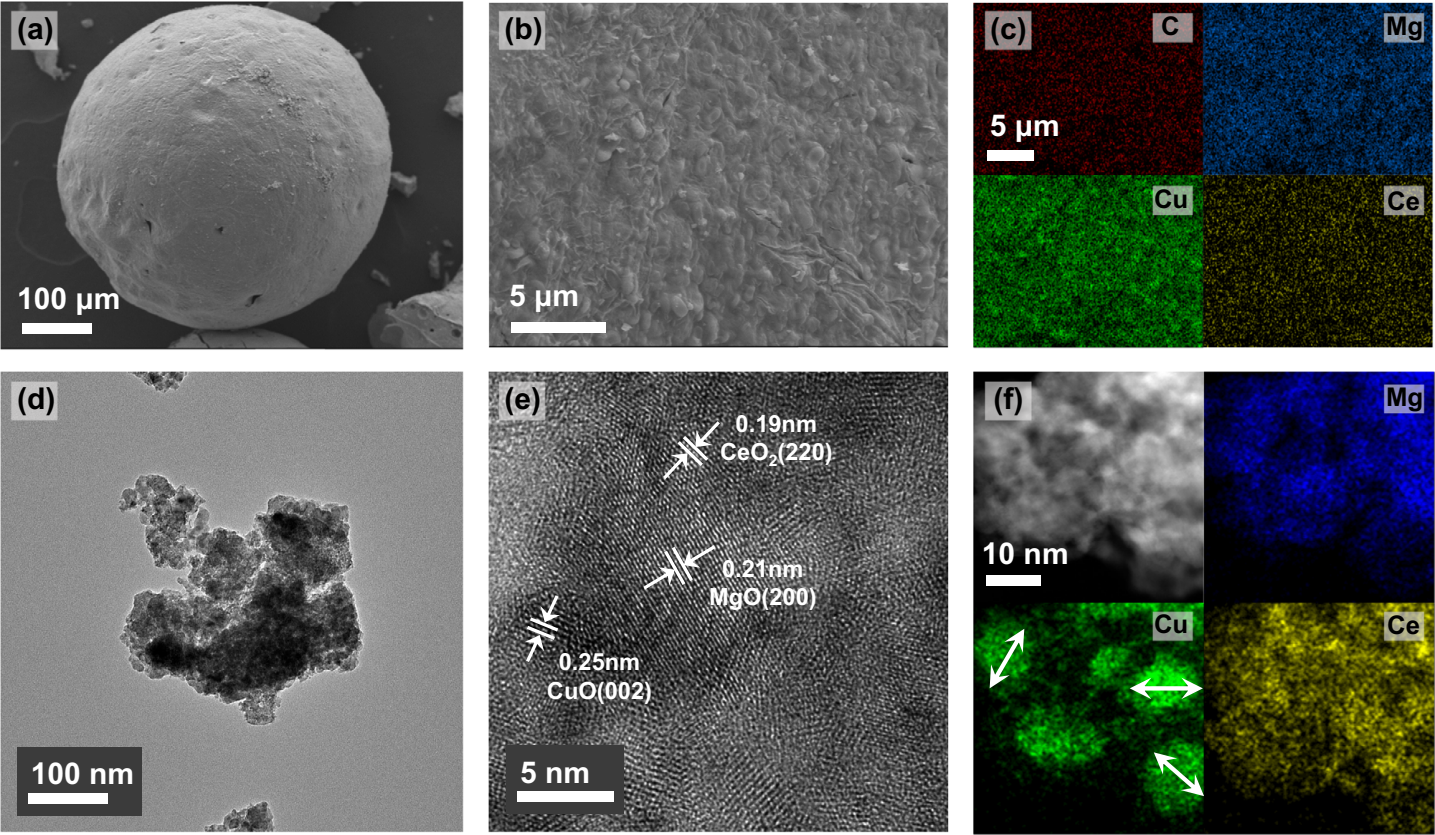
Structural analysis of Mg_13_CuCeO_*x*_. **a**–**c** SEM and EDS images, (**d**) TEM image of the ground sample, (**e**) HR-TEM image, and (**f**) STEM and corresponding elemental mapping images.

The scanning electron microscopy (SEM) image in Fig. 1a, b shows that Mg_13_CuCeO_*x*_ has a spherical bead morphology with a smooth surface, and the photograph of the bulk sorbents is presented in Supplementary Fig. 3. A spherical bead structure was observed for MgCuCeO_*x*_ only when the Mg content was higher than 13 wt.%. Both Mg and Ce were essential to the formation of the spherical structure using sol-gel combustion as presented in literature^23,31^, which results in powdered CuCeO_*x*_. The element mapping results of the SEM image in Fig. 1c confirmed the uniform distribution of Mg, Cu, and Ce, with a small amount of carbon from citric acid remaining even after calcination. The transmission electron microscopy (TEM) image in Fig. 1d shows a porous region and metal crystals in the Mg_13_CuCeO_*x*_ sample. MgO, CuO, and CeO_2_ crystal planes were confirmed by measuring the interplanar spacing in the high-resolution transmission electron microscopy (HR-TEM) image (Fig. 1e).

**Fig. 2:**
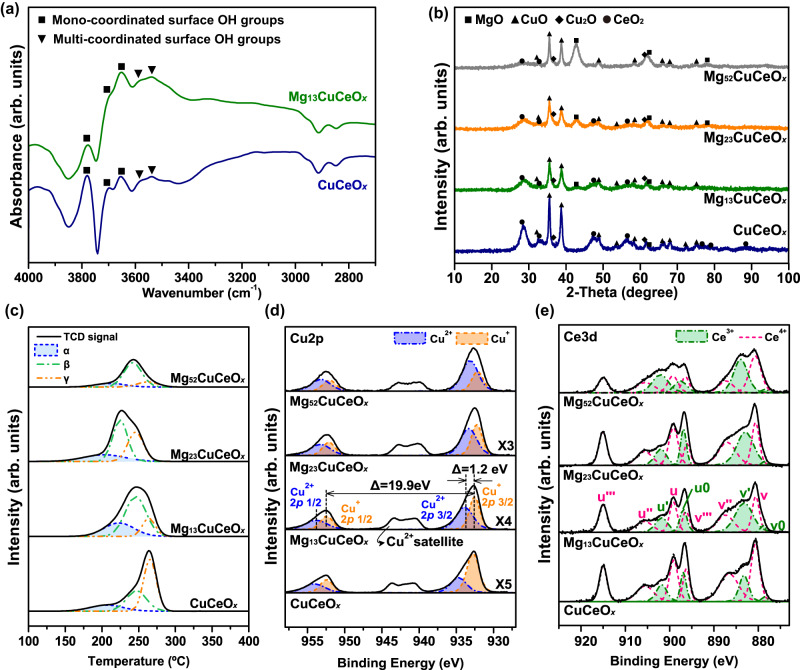
Physicochemical analysis of the as-prepared sorbent samples. **a** DRIFTS spectra; (**b**) XRD patterns; (**c**) H_2_-TPR results: The ratio of the area of α, a highly dispersed CuO_*x*_ species, to the combined area of (α + β + γ), signifying the total CuO_*x*_, is 16.6%, 20.3%, 27.5%, and 15.9%, from the topmost data downward, respectively; and XPS profiles: (**d**) Cu2*p* and (**e**) Ce3*d* spectra.

Figure 2a depicts the DRIFTS spectra of as-prepared Mg_13_CuCeO_*x*_ and CuCeO_*x*_ samples under He at 25 °C. Hydroxyl groups, bands within the range of 3300–3800 cm^−1^, were observed and consisted of multi-coordinated surface OH group (bands <3650 cm^−1^) and mono-coordinated surface OH groups (bands >3650 cm^−1^)^34^. Mg_13_CuCeO_*x*_ had a larger amount of OH groups. The introduction of Mg led to a significant increase in defects, resulting in the formation of numerous surface groups, especially, peaks at 3652, 3578, and 3536 cm^−1^^35^.

X-ray diffraction (XRD) diffractograms of MgO, CuO, Cu_2_O, and CeO_2_ were used to analyze the crystal structure of the as-prepared MgCuCeO_*x*_ and CuCeO_*x*_ samples (Fig. 2b). The CuO and CeO_2_ peaks became smaller and broader when Mg was introduced, indicating a reduction in the size of the crystallites. As shown in Fig. 2d, the as-prepared samples synthesized without a reduction step exhibited a reduction of Cu^2+^ to Cu^+^ in the range of 35–64%. The XRD patterns also revealed a relatively low intensity of Cu_2_O peaks, suggesting a high dispersion of Cu^+^ on the sorbent surface (Fig. 2b). The crystallite sizes of MgO, CuO, and CeO_2_ were calculated from the XRD patterns (Table 2). The calculated crystal size corresponded to the width of the individual CuO shown in the TEM-EDS image in Fig. 1f. The CuO and CeO_2_ crystals were both smaller in size within the MgCuCeO_*x*_ samples compared with the CuCeO_*x*_ samples. In particular, the size of the CeO_2_ crystals decreased as the Mg content increased, which was consistent with the results of a previous study^35^. Both Mg and Ce are known to contribute to the reduction in the crystal size of CuO^23,35^. Because the MgCuCeO_*x*_ samples were synthesized with a fixed Cu/Ce ratio in the present study, an increase in the Mg content reduced the Ce content (Table 2). It was found that the optimal level of Mg that minimized the crystallite size of CuO (10.7 nm) was 13 wt.%.

**Table 2:** Physicochemical properties of the as-prepared sorbent samples

Sorbents	Composition^a^ (wt. %)			Crystallite size^b^ (nm)			Peak area (%)			Cu dispersion (%)^e^	${S}_{{{{{{{\rm{Cu}}}}}}}^{+}}$^a, d,e^ (m^2^ g^−1^)
	Mg	Cu	Ce	MgO	CuO	CeO_2_	$\frac{\alpha }{(\alpha+\beta+\gamma )}$^c^	$\frac{{{{{{{\rm{Ce}}}}}}}^{3+}}{({{{{{{\rm{Ce}}}}}}}^{4+}+{{{{{{\rm{Ce}}}}}}}^{3+})}$^d^	$\frac{{{{{{{\rm{Cu}}}}}}}^{+}}{({{{{{{\rm{Cu}}}}}}}^{2+}+{{{{{{\rm{Cu}}}}}}}^{+})}$^d^		
CuCeO_*x*_	-	57.8	28.1	-	16.7	4.5	15.9	23	64	3.7	9.2
Mg_13_CuCeO_*x*_	10.6	45.5	22.0	4.2	10.7	2.6	27.5	40	46	14.7	21.0
Mg_23_CuCeO_*x*_	16.5	35.9	17.3	4.9	11.1	2.3	20.3	37	44	13.4	14.2
Mg_52_CuCeO_*x*_	33.3	20.2	9.6	4.7	13.0	0.9	16.6	43	35	11.0	5.2

^a^Estimated composition of Mg, Cu, and Ce ions by ICP-OES.

^b^Calculated from the XRD patterns by the Scherrer formula.

^c^Estimated from TPR results, and α indicates highly dispersed CuO species.

^d^Estimated from XPS results.

^e^Determined from N_2_O chemisorption.

The peaks in the temperature-programmed reduction using H_2_ (H_2_-TPR) results (Fig. 2c) were deconvoluted into highly dispersed CuO_*x*_ species (peak α), CuO_*x*_ species interacting with the support metals (peak β), and the bulk CuO_*x*_ phase (peak γ)^36^. Bulk CuO_*x*_ was predominantly observed in the CuCeO_*x*_ sample (peak γ, 52%), while the MgCuCeO_*x*_ samples consisted mainly of easily reducible Cu species (peaks α and β), with the largest area observed for Mg_13_CuCeO_*x*_.

The influence of the Mg content on *S*_Cu+_ (Supplementary Equation (3)) and the chemical states of Cu and Ce species was investigated by combining XPS and N_2_O chemisorption analysis (Fig. 2d, e, and Table 2). Because Cu^+^ ions allow both π-complexation and σ-bonding for CO sorption^19^, *S*_Cu+_ can be considered an indicator of CO sorbent performance. *S*_Cu+_ of CuCe-based sorbents is generally influenced by the Cu content, Cu dispersion, and Ce^3+^ ratio. The presence of Mg enhanced the surface Cu ratio for CuCeO_*x*_ (i.e., Cu dispersion, Table 2). In addition, the Ce^3+^ ratio (Fig. 2e and Table 2) improved with an increase in the Mg content, which can slow down the oxidation of Cu^+^ via the redox reaction Cu^+^ + Ce^4+^ ↔ Cu^2+^ + Ce^3+^. Because the samples were prepared using a fixed Cu/Ce ratio when adding Mg, an increase in the Mg content resulted in a decrease in the Cu content and the surface Cu^+^ ratio in the MgCuCeO_*x*_ samples (Fig. 2d and Table 2); despite this, the presence of Mg enhanced Cu dispersion and *S*_Cu+_. In the present study, the highest *S*_Cu+_ with the greatest Cu dispersion was observed for Mg_13_CuCeO_*x*_ (Table 2).

### CO sorption performance and dynamic behavior at trace levels

**Fig. 3:**
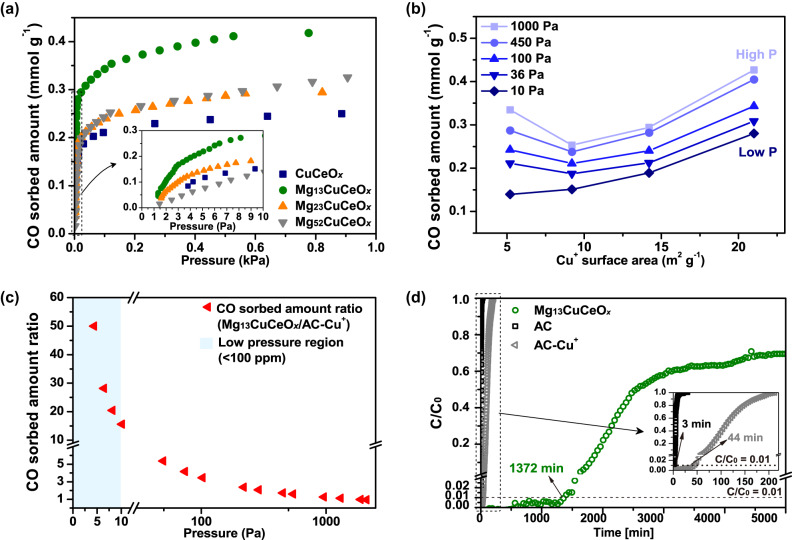
Analysis of CO sorption performance. **a** CO sorption isotherms for the as-prepared sorbent samples at 25 °C, (**b**) a comparison of CO sorbed amount of the sorbent as a function of Cu^+^ surface area at different pressures, (**c**) CO sorbed amount ratio between Mg_13_CuCeO_*x*_ and AC-Cu^+^, and (**d**) CO breakthrough curves at 25 °C under a flow of 50 ppm CO and H_2_ balance (GHSV = 935 h^−1^).

CO sorption isotherms recorded at 25 °C for the samples are presented in Fig. 3a. The isotherm curves exhibited a steep increase in the low-pressure range (≤10 Pa). The results indicated that MgCuCeO_*x*_ had a strong affinity for CO, which is an important characteristic for the purification of H_2_ to achieve fuel-cell-grade CO ppm levels. At 10 Pa (≈100 ppm CO at a total pressure of 100 kPa), the sorption capacity of the samples followed the order Mg_13_CuCeO_*x*_ > Mg_23_CuCeO_*x*_ > CuCeO_x_ > Mg_52_CuCeO_*x*_, which was consistent with *S*_Cu+_ (Table 2). However, at higher pressures (>90 Pa), Mg_52_CuCeO_*x*_, which had the highest surface area (Table 1), exhibited higher sorption than CuCeO_*x*_, with a similar isotherm to Mg_23_CuCeO_*x*_. Figure 3b depicts the relationship between *S*_Cu+_ and CO sorption capacity. At 10 Pa CO, the pressure range interested in this study, the sorption capacity displayed a positive correlation with increasing *S*_Cu+_. It can be deduced that *S*_Cu+_ is a critical determinant of sorption capacity under low CO concentration. Thus, our observations clearly indicated the necessity of well-dispersed Cu^+^.

Numerous sorbents had reported considerable sorption performance, but powder sorbents often pose problems in packed or fluidized bed applications due to their associated high-pressure drop and potential for process contamination^16^. Thus, given these factors, a pellet Cu-impregnated activated carbon (AC-Cu^+^), with outstanding performance among the reported CO sorbents^37^, served as a control group, and the performance evaluation incorporated both static and dynamic behavior. The CO sorption capacity of Mg_13_CuCeO_*x*_ was also compared with that of AC-Cu^+^ (Fig. 3c and Supplementary Fig. 4), sorbent with an excellent CO sorption capacity of 3.01 mmol g^−1^ at 100 kPa^37^. A breakthrough experiment was conducted to evaluate the dynamic sorption performance of pristine activated carbon (AC), AC-Cu^+^_,_ and Mg_13_CuCeO_*x*_ samples using 50 ppm CO (H_2_ balance) as the feed gas (Fig. 3d). The breakthrough time was determined at C/C_0_ = 0.01 (0.5 ppm CO), marking the red dotted line in the figure. Given the purified H_2_ mix from 0 minutes to the breakthrough time, its overall CO content is less than 0.2 ppm, and the sorbent should be regenerated after the breakthrough time. The detection times of 0.5 ppm CO in AC and AC-Cu^+^ beds were specified in the insert figure, with the time for the Mg_13_CuCeO_*x*_ bed marked in green. The dynamic sorption capacity was calculated as the sorption amount up to the saturation time at C = C_0_ (*S*_ads_(ST)) or a long-term duration of 5750 min (*S*_ads_(5750)), thereby providing comparative data for understanding sorption kinetic effects in practical applicability.

Compared to AC-Cu^+^, Mg_13_CuCeO_*x*_ exhibited 15.5 to 50 times higher sorption capacity at pressures below 10 Pa CO (Fig. 3c). As the pressure increased, this performance gap gradually diminished and ultimately inverted after 1700 Pa CO (Supplementary Fig. 4). These results suggested that Mg and Ce enhanced the CO sorption performance of Cu in the ultra-low CO pressure region.

As presented in Fig. 3d, Mg_13_CuCeO_*x*_ exhibited the highest performance with a breakthrough time of 1372 min compared to 3 min for AC and 44 min for AC-Cu^+^, indicating 31 times longer in terms of the breakthrough time. In addition, *S*_ads_(5750) of 0.209 mmol g^−1^ was also approximately 30 times higher than *S*_ads_(ST) of AC-Cu^+^. *S*_ads_(5750) for Mg_13_CuCeO_*x*_ was 75% of the equilibrium sorption amount, although the contact time between the sample and CO was limited during dynamic operation. Given that *S*_ads_(ST) of AC-Cu^+^ was only 39% of the equilibrium capacity, Mg_13_CuCeO_*x*_ demonstrated a relatively rapid sorption rate during the initial breakthrough period. A concentration plateau was observed in the breakthrough curve for Mg_13_CuCeO_*x*_ due to chemisorption, while it steadily sorbed a certain amount of CO even after 5750 min. Consequently, Mg_13_CuCeO_*x*_ has the potential to be successfully employed for the removal of low concentrations of CO even under dynamic conditions.

### Stability of cyclic CO sorption using oxidative regeneration

**Fig. 4:**
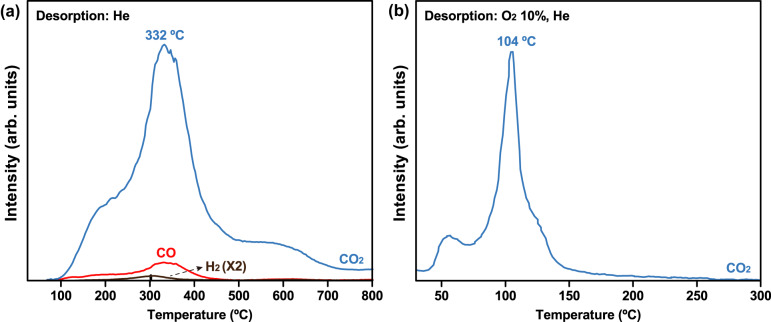
CO-TPD profiles for Mg_13_CuCeO_*x*_. CO-TPD profiles under (**a**) He and (**b**) O_2_ 10% (He balance).

The effective regeneration of a sorbent is a key factor in practical applications because this process typically requires significant energy and capital resources. In order to determine the regeneration conditions required for the repeated use of Mg_13_CuCeO_*x*_, its desorption properties under an inert gas (He) were assessed using CO-TPD analysis (Fig. 4a). The CO species chemisorbed onto the sorbent surface were mainly desorbed as CO_2_ at >100 °C, with a small quantity of CO released between 100 °C and 400 °C. It indicated that the strongly chemisorbed CO begins to be desorbed in the form of CO_2_ at elevated temperatures, instead of ambient temperatures. This phenomenon was corroborated by an additional breakthrough test (Supplementary Fig. 5). At 25 °C, any CO_2_ molecules were not observed until the breakthrough curve reached the feed concentration, further emphasizing that CO was being chemisorbed without concurrent emission of CO_2_. Furthermore, the absence of CO_2_ at ppb levels during the breakthrough tests indicated that the MgCuCeO_*x*_ sorbent can satisfy the fuel-cell grade hydrogen quality with a successful trace CO and without CO_2_ generation during a H_2_ purification step. Consequently, the MgCuCeO_*x*_ sorbent functioned as a CO_2_-free CO sorbent, rather than a catalyst for CO conversion, during CO removal step at ambient temperatures.

Trace H_2_ was detected at around 330 °C, which was associated with the interaction of CO with the OH groups on the sorbent surface (Fig. 2a). CO_2_ peaks were observed at high temperatures (200, 330, and 580 °C), representing a similar trend to that observed for the α, β, and γ peaks of Mg_13_CuCeO_*x*_ in the TPR analysis (Fig. 2c). The CO_2_ peaks at these temperatures originated from the desorption of CO species from the highly dispersed CuO_*x*_, CuO_*x*_ species interacting with the support metals, and the bulk CuO_*x*_ phase, respectively. The observed desorption behavior can be ascribed to either the disproportionation reaction (2CO → CO_2_ + C)^38^ or desorption with oxygen on the surface of a metal oxide (CO + O_sorbent_ → CO_2_)^39^, with both pathways potentially leading to the deterioration of the sorbent. In order to address this issue, the present study employed oxidative regeneration on the CO sorbent. Utilizing oxygen as the regeneration gas, degradation reactions can be suppressed^40,41^. It should be noted that the energy requirement for the CO + O_regeneration_ → CO_2_ reaction is considerably lower than that for the aforementioned degradation reactions.

Figure 4b presents the CO-TPD results when oxygen (O_2_ 10%, He balance) was employed as the regeneration gas. Using oxidative regeneration, the temperature required for maximum desorption was significantly lower at 104 °C compared with 332 °C under inert gas flow. Additionally, desorption occurred only in the form of CO_2_. Consequently, employing oxidative regeneration represented an effective energy-efficient solution to sorbent deterioration.

**Fig. 5:**
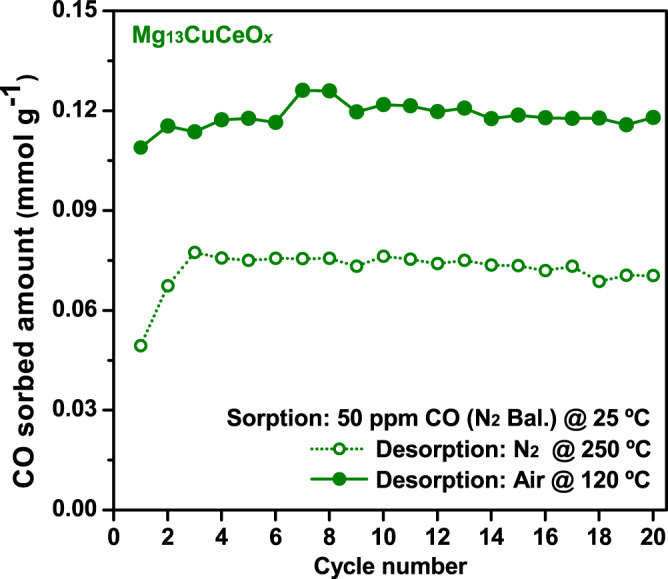
Cyclic sorption-regeneration tests for Mg_13_CuCeO_*x*_. Cyclic tests with sorption for 60 min at 25 °C under 50 ppm CO (N_2_ balance) and regeneration for 30 min in air (21% O_2_ and N_2_ balance) at 120 °C or in N_2_ at 250 °C.

Figure 5 presents the results of the stability test of Mg_13_CuCeO_*x*_ over 20 cycles following 30-min pretreatment with air or N_2_ gas at 450 °C. During the sorption process, 50 ppm CO (N_2_ balance) was employed, while air or N_2_ was used as the desorption medium. The sorption and regeneration periods were 1 h and 30 min, respectively. The samples pretreated and regenerated using air exhibited a cyclic working capacity of 0.12 mmol g^−1^, efficiently sorbing most of the CO present (>99%) with excellent stability. These findings indicated that the Cu^+^ ions were conserved after oxidative regeneration, and this was corroborated by the XPS (Fig. 2d) and *S*_Cu+_ results (Table 2) obtained immediately after sorbent synthesis using air calcination.

In contrast, the sample pretreated with N_2_ experienced a decline in its sorption capacity of approximately 50% compared with air pretreatment, and a progressive reduction in its sorption capacity was observed with each successive sorption–desorption cycle, which was attributed to a lower sorption rate. This hypothesis is supported by previous studies reporting the impact of O_2_ pre-adsorption on the sorption–oxidation rate of CO^42,43^. Overall, even when air, which is significantly more cost-effective and readily available than reducing or inert gases, was utilized for pretreatment or regeneration, the Cu^+^ ions on the MgCuCeO_*x*_ surface remained intact and the sorption rate remained high.

### Mechanistic study of CO chemisorption

**Fig. 6:**
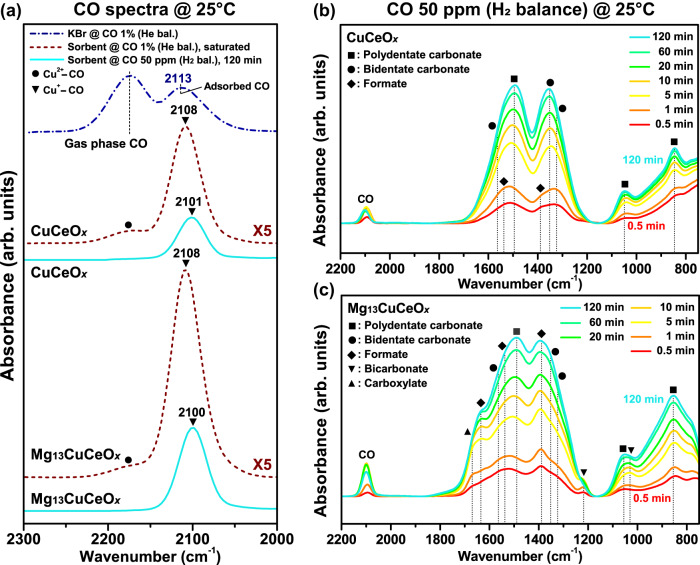
DRIFTS Spectra Comparison. (**a**) DRIFTS spectra comparison. (**a**) DRIFTS spectra of CO sorbed on Mg_13_CuCeO_*x*_ and CuCeO_*x*_ at different CO compositions and In-situ DRIFTS spectra for (**b**) CuCeO_*x*_ and (**c**) Mg_13_CuCeO_*x*_ at 25 °C in 50 ppm CO (H_2_ balance).

CO DRIFTS spectra of CuCeO_*x*_ and Mg_13_CuCeO_*x*_ under 50 ppm CO and 1% CO were compared with gaseous CO peaks of inert KBr (without a sorbent sample) under 1% CO (Fig. 6a). Under 1% CO, both Mg_13_CuCeO_*x*_ and CuCeO_*x*_ exhibited peaks at 2174 and 2108 cm^−1^, associated with Cu^2+^–CO and Cu^+^–CO, respectively^44^. As shown in the magnified view of Fig. 6a in Supplementary Fig. 7, the band intensity ratio of the Cu^+^–CO to Cu^2+^–CO band was larger in Mg_13_CuCeO_*x*_. This indicated that Mg increases the surface area of Cu^+^, as shown in Table 2, enhancing its accessibility to CO (Fig. 3). Furthermore, the bands, corresponding to Cu^+^–CO of CuCeO_*x*_ and Mg_13_CuCeO_*x*_, exhibited a red shift compared to gaseous CO, and this shift was larger in 50 ppm CO case than in 1% CO case. The band shift for Cu^+^–CO can result from the contribution of σ and π bonds. The red shifts implied the dominance of π bonding during CO sorption onto the samples^20^, which was amplified at lower pressures.

In-situ DRIFTS was employed to investigate the CO sorption mechanisms at extremely low pressures and to elucidate the role of Mg in the sorbent. In-situ DRIFTS spectra were recorded for the Mg_13_CuCeO_*x*_ and CuCeO_*x*_ samples during the breakthrough experiment using 50 ppm CO gas (H_2_ or N_2_ balance) at 25 °C (Fig. 6b, c and Supplementary Fig. 6). The peak assignments and structure of the analogous carbonates produced in the present study are presented in Supplementary Table 2. For CuCeO_*x*_ (Fig. 6b), polydentate carbonate (1492, 1052, and 850 cm^−1^, indicated by the square symbol) and bidentate carbonate (1563, 1350, and 1323 cm^−1^, indicated by the circle symbol) were the predominant sorbed CO species for both H_2_ and N_2_ as the balance gas. These species became dominant after 5 min, which was consistent with the carbonate species that formed during chemisorption on the surface of a CuO-based commercial catalyst^45^. Minor contribution of CO physisorption was evidenced by the CO peak at 2100 cm^−1^, which was saturated after about 60 min. Additionally, trace peaks for formate species (1392 and 1535 cm^−1^, indicated by the diamond symbol) were observed within 1 min.

During CO chemisorption on Mg_13_CuCeO_*x*_ (Fig. 6c), polydentate carbonate and bidentate carbonate were observed as the main peaks, and other routes were identified, which could be attributed to the presence of Mg. Noticeably, formate species, which were indicated by the peaks at 1635, 1535, and 1392 cm^−1^ (υ_as_(COO)^46^, υ_s_(OCO)/υ_as_(OCO)^47^, and C–H bending^48^, respectively), were a key pathway. A carboxylate peak at 1670 cm^−1^ and bicarbonate peaks at 1218 and 1038 cm^−1^ were also observed as secondary peaks. The bicarbonate peak at 1218 cm^−1^, corresponding to υ(CO_3_), increased until 10 min and then decreased, indicating that bicarbonate acts as an intermediate during the chemisorption process. Interestingly, the formation and disappearance of bicarbonate occurred more slowly when N_2_ was used as the balance gas (Supplementary Fig. 6b), suggesting that the presence of H_2_ in the feed can accelerate the bicarbonate-related chemisorption process. The observation that bicarbonate (HCO_3_^−^) and formate (HCO_2_^−^) species were exclusively formed on Mg_13_CuCeO_*x*_ even in the presence of the N_2_ balance gas suggested that the OH groups on the sorbent surface played a significant role in the formation of these species. The chemisorption mechanisms for Mg_13_CuCeO_*x*_ were thus assumed to involve an additional pathway associated with the surface OH groups, leading to the rapid formation of bicarbonate (HCO_3_^−^) species during the initial stages of the chemisorption process, followed by their conversion into formate (HCO_2_^−^).

**Fig. 7:**
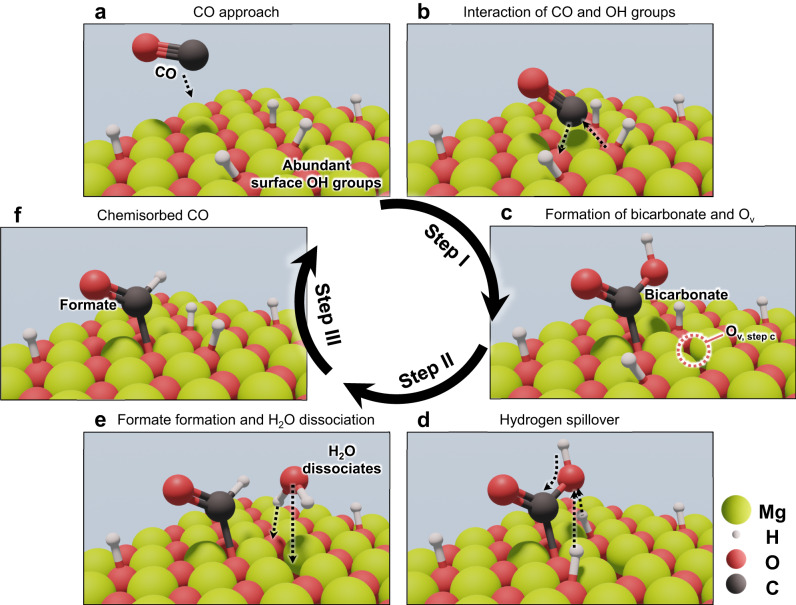
Schematic diagram of the OH group-based CO chemisorption pathway on MgCuCeO_*x*_. (**a**) CO approach, (**b**) interaction of CO and OH groups, (**c**) formation of bicarbonate and oxygen vacancy (O_v_), (**d**) hydrogen spillover, (**e**) formate formation and water dissociation, and (**f**) chemisorbed CO.

Based on the spectral analysis, a possible catalytic reaction pathway for CO on the surface of MgCuCeO_*x*_, was proposed (Fig. 7). In Step I (Fig. 7a–c), a CO molecule interacts with an OH group on the sorbent surface. This interaction is facilitated by the weakly basic OH groups, which promote the generation of bicarbonate species^49^. When CO is introduced to the MgCuCeO_*x*_ surface, it acquires an additional oxygen atom from the surface, leading to the creation of an oxygen vacancy (O_v, step c_) and bicarbonate. In Step II (Fig. 7d, e), the bicarbonate species reacts with H atoms from neighboring surface OH groups to generate water and formate, as reported by previous studies^50^. In Step III (Fig. 7f), the water dissociates into OH and H at the O_v, step c_, and surface oxygen, respectively^34^, thus regenerating OH groups. The presence of Mg in the sorbent contributes to the formation of surface OH groups (Fig. 2a), playing a crucial role in this CO chemisorption pathway and enhancing the sorption capacity of the sorbent. Consequently, Mg_13_CuCeO_*x*_ exhibited high CO removal performance with excellent stability in the cyclic test (Fig. 5).

## Discussion

We successfully developed a highly stable bead-structured Mg, Cu, and Ce-based sorbent for the effective removal of trace CO from H_2_ gas. By including Mg in the CuCeO_*x*_-based sorbent, we achieved a pore-rich bead structure and a large surface area. The optimal Mg ratio was found to enhance the dispersion by producing smaller Cu crystals, which was essential to the superior sorption performance. MgCuCeO_*x*_ retained a high Cu^+^ surface area even after air calcination, which was attributed to the presence of Mg increasing the proportion of Ce^3+^ to promote the redox reactions. Mg_13_CuCeO_*x*_ exhibited superior CO sorption at both equilibrium and under dynamic conditions at ultra-low pressures below 10 Pa (≈100 ppm CO at a total pressure of 100 kPa), a performance that surpassed that of previously reported pelletized sorbents. This suggests that Mg_13_CuCeO_*x*_ can be efficiently employed as a sorbent for the production of fuel-cell-grade H_2_.

Moreover, we demonstrated oxidative regeneration as a viable strategy for the repeated use of a sorbent. The pre-adsorption of oxygen had a beneficial influence on the reaction rate during the CO sorption–oxidation process for MgCuCeO_*x*_. This ensured that Mg_13_CuCeO_*x*_ maintained its sorption capacity during cyclic tests, highlighting its stability and the effectiveness of air for pretreatment and regeneration. We also showed that oxidative regeneration is an energy-efficient method without any sorbent degradation.

We explored the CO chemisorption mechanisms associated with MgCuCeO_*x*_ using in-situ DRIFTS, highlighting the critical role of Mg in enhancing the sorption capacity. Mg increased the abundance of surface OH groups, which became active centers in the catalytic reactions for the formation of bicarbonate and formate species. This process ensured continuous CO removal and demonstrated the importance of incorporating Mg into CuCeO_*x*_-based sorbents. Therefore, in the breakthrough test, Mg_13_CuCeO_*x*_ continuously removed a certain amount of CO even after 5000 min of exposure to a 50 ppm CO stream at a GHSV of 935 h^−1^.

Consequently, the presence of Mg contributed to improving the structural and chemical properties of the sorbent, thus enhancing its sorption capacity and cyclic stability. Therefore, MgCuCeO_*x*_ represents a promising, effective, and stable sorbent for ultra-low levels of CO, one that does not discharge CO_2_ during the removal process. Since MgCuCeO_*x*_ proved a high removal potential of trace CO in H_2_ and was readily regenerated at 120°C under an air flow, simple systems using MgCuCeO_*x*_ can cost-effectively remove trace CO. These results highlight the potential of this sorbent for practical applications targeting the generation of ultra-high-purity H_2_, especially for use in fuel cells.

## Methods

### General

All reagents were purchased from vendors and used without further purification. The structural properties were acquired from N_2_ adsorption/desorption isotherms at 77 K with an Autosorb IQ instrument (Quantachrome, version 5.21). The morphology and elemental distribution were examined by a combination of FE-SEM, TEM, and EDS techniques using JSM-7610F-Plus (JEOL, Ltd.), JEM-ARM 200 F (NEOARM), (JEOL, Ltd.), and X-MAX TSR (OXFORD Instruments), respectively. X-ray diffraction (XRD) data was collected from an Ultima IV diffractometer (Rigaku) with Cu-Kα radiation (λ = 1.54 Å). The H_2_-TPR measurement was carried out using a ChemBET Pulsar TPR/TPD unit (Quantachrome). XPS analyses were performed using Thermo Fisher Scientific with monochromated Al Kα radiation as the excitation source. Cu dispersion and the S_Cu_ were determined via selective N_2_O chemisorption experiments conducted at 50 °C.

### Sorbent synthesis

MgCuCeO_*x*_ beads and CuCeO_*x*_ powders were synthesized using a sol-gel combustion-assisted method. Magnesium nitrate hexahydrate (0.01 mol) with the desired amount of copper nitrate trihydrate, cerium nitrate hexahydrate, and citric acid monohydrate was dissolved in deionized water. The detailed ratios of each substance are described in the Supplementary Methods. The solution was then stirred in an oil bath at 80 °C for 5 h and dried at 90 °C for 2 h. The dried sample was ground and sieved (mesh size of 250–600 μm), followed by drying overnight at 110 °C. Lastly, the resulting sample was calcined under a flow of 21% O_2_ (with an N_2_ balance, hereafter referred to as air for simplicity) by ramping the temperature to 450 °C at a heating rate of 1 °C min^−1^ and maintained for 10 h.

AC-Cu^+^ was synthesized using the impregnation method^37^. Pristine AC support was pre-treated at 700 °C for 3 h under H_2_ flow. The pre-treated AC was then impregnated with 4.26 mmol of copper per gram of sorbent by stirring for 1 h at 60 °C in a solution of copper formate tetrahydrate and HCl. The impregnated sorbent was then washed, filtered, and dried at 60 °C overnight. The final AC-Cu^+^ was obtained by activating the dried sorbent under N_2_ flow at 100 mL min^−1^ and 300 °C for 3 h. The details of these experiments can be found in Supplementary Methods.

### CO sorption and desorption test

CO sorption isotherms were acquired using a commercial sorption analyzer (Autosorb IQ, Quantachrome, version 5.21) and the conventional static volumetric method under pressures of up to 1 kPa. Before analysis, the CuCeO_*x*_ and MgCuCeO_*x*_ samples underwent vacuum degassing at 280 °C for 8 h to eliminate adsorbed impurities.

Prior to the breakthrough experiment, AC was treated at 120 °C for over 12 h under He flow, AC-Cu^+^ was treated at 200 °C for 3 h, and Mg_13_CuCeO_*x*_ was treated with air at 280 °C for 30 min. The breakthrough apparatus (Supplementary Fig. 1) with a CO-infrared analyzer with a detection limit of 0.02 ppm (CO-IR, Everise) was utilized to perform breakthrough tests. All experiments were conducted at 1 bar, 25 °C, and a GHSV of 935 h^−1^.

The desorption behavior of Mg_13_CuCeO_*x*_ was examined using CO-TPD with a Chem BET Pulsar TPR/TPD unit in conjunction with an online mass spectrometer (MS; HPR 20, Hiden Analytical Ltd.). Following chemisorbent pretreatment under the same conditions as for the breakthrough experiment, the temperature was reduced to 25 °C and the samples were purged with He. The samples were then exposed to 1% CO (He balance) at a flow rate of 50 ml min^−1^ at 25 °C for 1 h to reach saturation. A He purge was then conducted until no residual gas was detected by the MS. The regeneration of the chemisorbent samples was then conducted using either He or air under heating up to 800 °C.

The cyclic CO uptake of Mg_13_CuCeO_*x*_ was investigated using TGA (TGA 4000, Perkin Elmer) at 1 bar. Prior to each experiment, the Mg_13_CuCeO_*x*_ sample was subjected to pretreatment at 450 °C for 30 min under air or N_2_ (40 mL min^−1^). Cyclic sorption was conducted with the removal step occurring under a flow of 50 ppm CO (N_2_ balance, 40 mL min^−1^) at 25 °C for 60 min and the regeneration step under air at 120 °C or N_2_ at 250 °C (40 mL min^−1^) for 30 min. The details of these experiments can be found in Supplementary Methods.

### In-situ DRIFTS experiments

In-situ IR spectra were collected using a Nicolet iS10 FT-IR instrument equipped with a DRIFT accessory and a cell from PIKE Technologies. The spectra were recorded with a resolution of 4 cm^−1^ and 64 scans, and an MCT cryodetector was used for signal detection. The samples were pretreated under air with a flow rate of 50 mL min^−1^ at 450 °C for 30 min using a high-temperature cell equipped with Zn-Se windows. The background IR spectrum was measured at the sorption temperature of 25 °C before exposing the pretreated samples to 50 ppm CO (H_2_ or N_2_ balance) in a feed stream with a flow rate of 50 mL min^−1^ at 25 °C for 120 min.

### Supplementary information

Supplementary Information

Peer Review File

## Data Availability

The data to support the findings of this study are available from the corresponding authors upon request.
