# Supplementary material for: Mg-incorporated sorbent for efficient removal of trace CO from H2 gas
Source: Nat Commun. 2023 Nov 3;14:7045. doi: 10.1038/s41467-023-42871-6 (PMC10624860; doi:10.1038/s41467-023-42871-6)
Supplement: Supplementary file 1 — Supplementary Information [file 41467_2023_42871_MOESM1_ESM.pdf]

**Supplementary Information *(Nature Communications)***

# Mg-incorporated sorbent for efficient removal of trace CO from $H_2$ gas

Gina Bang,[1] Seongmin Jin,[2] Hyokyung Kim,[1] Kyung-Min Kim[3]* and Chang-Ha Lee[1]*

[1] Department of Chemical and Biomolecular Engineering, Yonsei University, Seoul, Republic of Korea

[2] Institute of Chemical Sciences and Engineering, École Polytechnique Fédérale de Lausanne (EPFL), Lausanne, Switzerland

[3] Department of Biochemical Engineering, Gangneung-Wonju National University, Gangneung, Republic of Korea

## Corresponding Authors

*K.M.K kmkim@gwnu.ac.kr

*C.H.L. leech@yonsei.ac.kr

**This file includes:**

**Supplementary Methods**

**Supplementary Figures 1-7**

**Supplementary Tables 1-2**

**Supplementary References**

## Supplementary Methods

### Materials

The study utilized magnesium nitrate hexahydrate (Sigma-Aldrich, India, 99.0%), copper nitrate trihydrate (Sigma-Aldrich, Spain, 99.0%), cerium nitrate hexahydrate (Sigma-Aldrich, France, 99.0%), and citric acid monohydrate (Sigma-Aldrich, USA, 98.0%) as received without any additional purification. Petroleum-based activated carbon (AC) beads were obtained from Kureha Corp. and impregnated with Cu for a CO removal comparison (AC-$Cu^{+}$). Copper formate tetrahydrate (Alfa Aesar Chemicals, 99%) and hydrochloric acid (Daejung Chemicals & Metals, assay 35%) were also used without further purification. For the sorption and regeneration tests, $N_2$ (Daedeok, Korea, 99.999%), CO (Air Liquide, Korea, 99.95 %), $H_2$ (Daedeok, Korea, 99.999 %), and He (Daedeok, Korea, 99.999%) were employed. Furthermore, 50 ppm CO gas with an $N_2$ or $H_2$ balance were purchased from Union Gas, Korea.

### Sorbent synthesis

Powdered $CuCeO_x$ and $MgCuCeO_x$ beads were synthesized using a sol-gel combustion-assisted method. The ratio of Mg to total metal was 0.13, 0.23, and 0.52 (w/w), with a fixed Cu to Ce molar ratio of 4.4. Magnesium nitrate hexahydrate (0.01 mol) with the desired amount of copper nitrate trihydrate, cerium nitrate hexahydrate, and citric acid monohydrate was dissolved in deionized water. The molar ratio of citric acid and water to the metals (Mg, Cu, and Ce) was fixed at 2 and 60, respectively. For instance, $Mg_{13}CuCeO_x$ comprises 2.5641 g of magnesium nitrate hexahydrate, 3.9372 g of copper nitrate trihydrate, 1.6082 g of cerium nitrate hexahydrate, 32.4 ml of water, and 12.6084 g of citric acid. Based on these quantities, the weight ratios for Mg, Cu, and Ce are 0.13, 0.58, and 0.29, respectively.

The solution was then stirred in an oil bath at 80°C for 5 h and dried at 90°C for 2 h. The dried sample was ground and sieved (mesh size of 250–600 μm) to obtain a sample with a specific diameter range. The sample was then dried overnight at 110°C, resulting in a powder or spherical bead morphology depending on the precursor metal type and composition. Lastly, the resulting sample was calcined using a flow of 21% $O_2$ (with an $N_2$ balance, hereafter referred to as air for simplicity) by ramping the temperature to 450°C at a heating rate of 1°C $min^{-1}$ and maintained for 10 h. The final samples were denoted as $CuCeO_x$ and $Mg_{\alpha}CuCeO_x$, with $\alpha$ indicating the weight percentage of Mg compared with the total metal.

For comparison, pristine AC and AC-$Cu^+$, which exhibited superior CO sorption performance in a previous study [1], were prepared using the same method described in previous research. The pristine AC support was pre-treated at 700°C for 3 h under $H_2$ flow. The pre-treated AC was then impregnated with a solution of copper formate tetrahydrate and HCl, targeting a copper content of 4.26 mmol per 1 g of sorbent, and stirred at 60°C for 1 h. After impregnation, the sorbent was washed, filtered, and dried at 60°C overnight. The final AC-$Cu^+$ was prepared by activating the dried sorbent under $N_2$ flow at 100 mL $min^{-1}$ and 300°C for 3 h.

**Characterization**

The structural properties of the as-synthesized chemisorbent samples were assessed using $N_2$ adsorption/desorption isotherms acquired at 77 K with an Autosorb IQ instrument (Quantachrome, version 5.21). To examine the morphology and elemental distribution of the chemisorbents, a combination of field-emission scanning electron microscopy (FE-SEM; JSM-7610F-Plus, JEOL, Ltd.) and transmission electron microscopy (TEM; JEM-ARM 200F (NEOARM), JEOL, Ltd.) was employed. Elemental mapping was performed utilizing an energy-dispersive X-ray spectrometer (EDS; X-MAX TSR, OXFORD Instruments).

The crystalline structure of the as-prepared chemisorbent samples was assessed using X-ray diffraction (XRD) analysis conducted with an Ultima IV diffractometer (Rigaku), operating at 40 kV and 100 mA with Cu-Kα radiation (λ = 1.54 Å). XRD patterns were recorded over a 2θ range of 10° to 100°, with scanning increments of 0.02°. To determine the metal composition of the samples, inductively coupled plasma optical emission spectrometry (ICP-OES; 5110, Agilent) was employed.

The temperature-programmed reduction using $H_2$ ($H_2$-TPR) was carried out using a ChemBET Pulsar TPR/TPD unit (Quantachrome). For this, 50 mg of a sorbent sample was loaded into a U-shaped quartz reactor and subjected to reduction by heating from ambient temperature to 800°C at a heating rate of 10°C $min^{-1}$ under a 10% $H_2$/Ar gas flow. A cold trap was employed to capture water produced during the experiment before entering the thermal conductivity detector (TCD).

The surface chemistry of the samples was characterized using XPS (Thermo Fisher Scientific) with monochromated Al Kα radiation as the excitation source. The Ce3*d* spectra were subjected to baseline subtraction, after which a well-resolved u''' peak was identified at around 915 eV in the spectrum. The positions of the remaining peaks were determined by referencing the binding energy shift relative to the u''' peak [2]. The Cu2*p* spectra comprising $Cu2p^{3/2}$ and $Cu2p^{1/2}$ peaks were deconvoluted into $Cu^{+}$ and $Cu^{2+}$ components [3]. Finally, the ratios of $Ce^{3+}$ to Ce species and $Cu^{+}$ to Cu species were calculated based on their respective peak areas.

Cu dispersion and the specific Cu surface area ($S_{Cu}$) were determined using selective $N_2O$ chemisorption experiments conducted at 50°C following a well-documented methodology [4,5]. Additionally, since $Cu^{+}$ is the active site for the sorption of CO on $MgCuCeO_x$, the surface

area of $Cu^+$ of the as-prepared sorbent ($S_{Cu+}$) was estimated by applying the surface $Cu^+$ ratio to $S_{Cu}$. Initially, the samples were reduced under a flow of 10% $H_2$/Ar at 400°C for 1 h, and the $H_2$ consumption was measured by integrating the peak area. The chemisorbent bed was then cooled to 50°C and flushed with He. The cooled chemisorbent was exposed to 10% $N_2O$/He gas for 30 min before being returned to room temperature and purged with He. An additional $H_2$-TPR cycle was performed and the corresponding $H_2$ consumption was assessed. Cu dispersion (D) was calculated based on the $H_2$ consumption after the total oxidation of the catalyst in the first $H_2$-TPR run ($X$) and subsequent oxidation of surface Cu atoms in the second $H_2$-TPR run ($Y$). Cu dispersion and $S_{Cu}$ were determined using Supplementary Equations (1) and (2) respectively:

$$D = 2 \times Y/X \quad (1)$$

$$S_{\mathrm{Cu}} = (D \times N_{\mathrm{av}} \times W_{\mathrm{Cu}})/(A_{\mathrm{Cu}} \times 1.4 \times 10^{19}) \quad (2)$$

where $N_{av}$ is the Avogadro constant ($6.02 \times 10^{23}$ mol$^{-1}$), $W_{Cu}$ indicates the Cu metal weight content determined using ICP-OES analysis, and $A_{Cu}$ is the atomic weight of Cu (63.546 g mol$^{-1}$). The ratio of $Cu^+$ to $Cu^{2+}$ ($M$), obtained from the XPS analysis, was then used to calculate $S_{Cu+}$ by multiplying $S_{Cu}$ by $M$ (Supplementary Equation (3)).

$$S_{\mathrm{Cu^+}} = S_{\mathrm{Cu}} \times M \quad (3)$$

**CO sorption and desorption test**

CO sorption isotherms were acquired using a commercial sorption analyzer (Autosorb IQ, Quantachrome, version 5.21) and the conventional static volumetric method under pressures of up to 1 kPa. Before analysis, the $CuCeO_x$ and $MgCuCeO_x$ samples underwent vacuum degassing at 280°C for 8 h to eliminate adsorbed impurities.

For the breakthrough experiment, the chemisorbent samples were subjected to pretreatment to eliminate impurities or emulate the conditions immediately after synthesis. AC was treated under a stream of He at 120°C for over 12 h, AC-$Cu^+$ under He at 200°C for 3 h, and $Mg_{\alpha}CuCeO_x$ under air at 280°C for 30 min. The breakthrough apparatus (Supplementary Fig. 1) consisted of three mass flow controllers (MFCs; Bronkhorst Co.) to supply the gas, a resistance temperature detector (RTD, Pt 100 Ω) to control the temperature, a reactor with an internal diameter of 0.7 cm, and a CO-infrared analyzer with a detection limit of 0.02 ppm (CO-IR, Everise), details of which can be found in a previous study [1]. The MFCs were calibrated using a mass flow meter (Hastings Inc., USA) and a soap bubble flow meter (Supelco Co., USA). All experiments were conducted at 1 bar, 25°C, and a gas hourly space velocity (GHSV) of 935 $h^{-1}$.

The desorption behavior of $Mg_{13}CuCeO_x$ was examined using CO temperature-programmed desorption (CO-TPD) with a Chem BET Pulsar TPR/TPD unit in conjunction with an online mass spectrometer (MS; HPR 20, Hiden Analytical Ltd.). A U-shaped quartz reactor was used to contain the sample (0.05 g). Following chemisorbent pretreatment under the same conditions as for the breakthrough experiment, the temperature was reduced to 25°C and the samples purged with He. The samples were then exposed to 1% CO (He balance) at a flow rate of 50 ml $min^{-1}$ at 25°C for 1 h to reach saturation. A He purge was then conducted

until no residual gas was detected by the MS. The regeneration of the chemisorbent samples was then conducted using either He or air under heating up to 800°C.

The cyclic CO uptake of $Mg_{13}CuCeO_x$ was investigated using TGA (TGA 4000, Perkin Elmer) at 1 bar. Prior to each experiment, the $Mg_{13}CuCeO_x$ sample was subjected to pretreatment at 450°C for 30 min under air or $N_2$ (40 mL min$^{-1}$). Cyclic sorption was conducted with the removal step occurring under a flow of 50 ppm CO ($N_2$ balance, 40 mL min$^{-1}$) at 25°C for 60 min and the regeneration step under air at 120°C or $N_2$ at 250°C (40 mL min$^{-1}$) for 30 min.

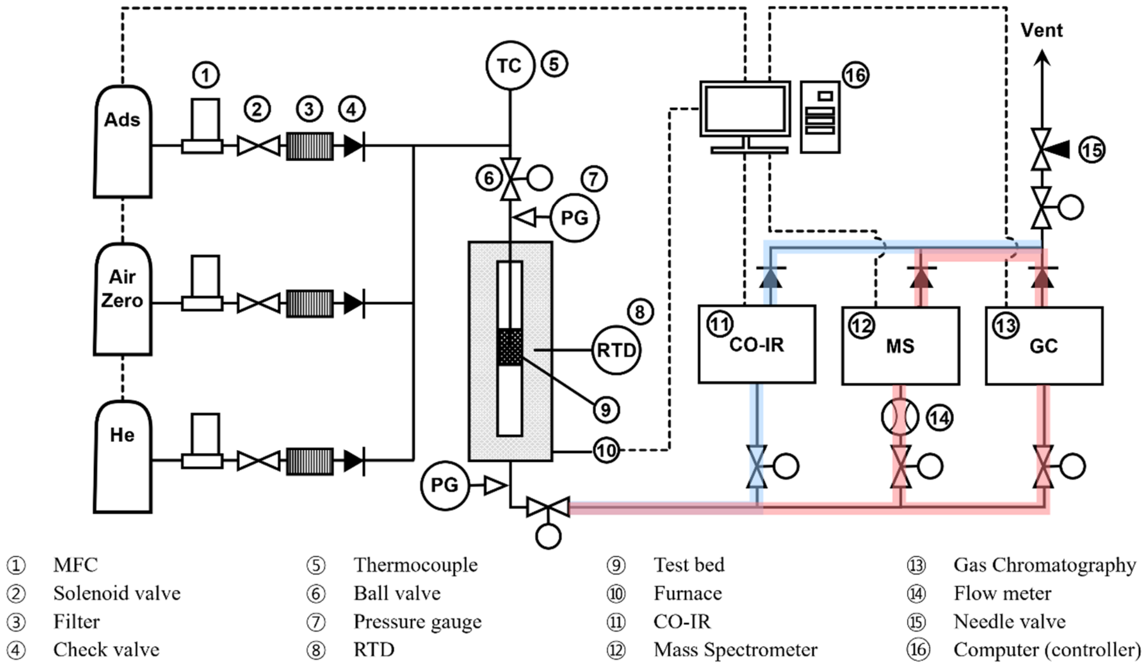


**Supplementary Figure 1.** Schematic diagram of breakthrough experimental apparatus.

In the long-term breakthrough experiment presented in Fig. 3d, we employed a monitoring approach illustrated by the blue line in Supplementary Fig. 1, which exclusively focused on CO detection via infrared (IR) spectroscopy. Conversely, the breakthrough experiment presented in Supplementary Fig. 5 was designed for the simultaneous detection of CO and $CO_2$ at ppb levels until the breakthrough curve reached the feed concentration. For this full breakthrough curve experiment, we used a more complex analysis setup that included a gas chromatograph (GC) equipped with a flame ionization detector (FID) and a methanizer, along with a mass spectrometer (MS). This experimental methodology is represented by the red line in Supplementary Fig. 1.

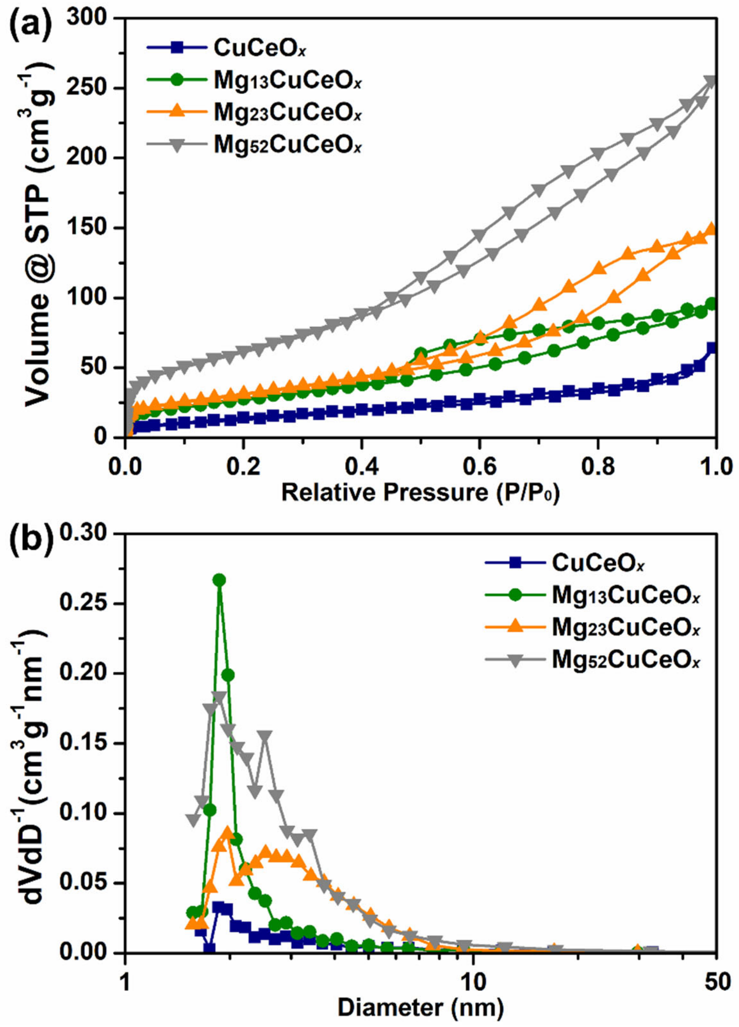


**Supplementary Figure 2.** (a) $N_2$ adsorption and desorption isotherms at 77 K and (b) pore size distribution calculated using the BJH method for the chemisorbent samples.

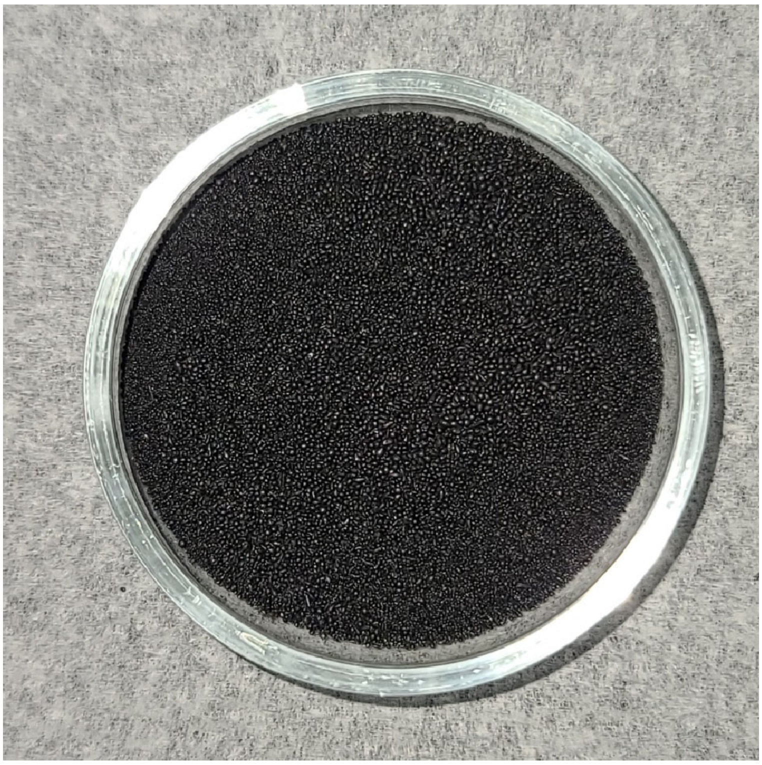

**Supplementary Figure 3.** Photograph of $MgCuCeO_x$ beads.

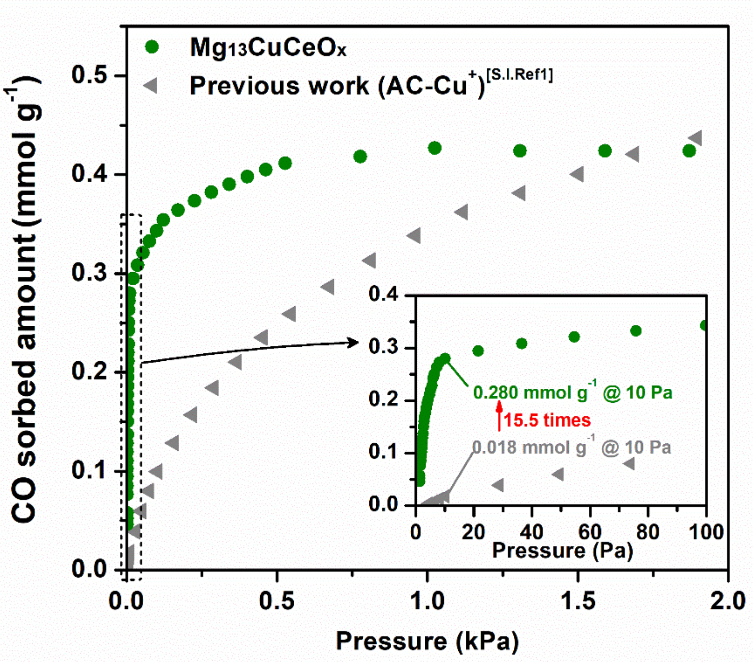


**Supplementary Figure 4.** CO sorption isotherms at 25°C for $Mg_{13}CuCeO_x$ and $AC\text{-}Cu^+$.

To the best of our knowledge, no previous studies have measured the sorption isotherm for CO in this ultra-low pressure region [6]. Therefore, we compared the CO sorption performance of $Mg_{13}CuCeO_x$ with the $AC\text{-}Cu^+$ sorbent recently reported in our previous study [1]. The $AC\text{-}Cu^+$ sorbent exhibited an excellent CO sorption performance of 3.01 mmol $g^{-1}$ at 100 kPa. The data presented in Supplementary Fig. 4 were newly generated in the present study because the previous study did not include the low-pressure region.

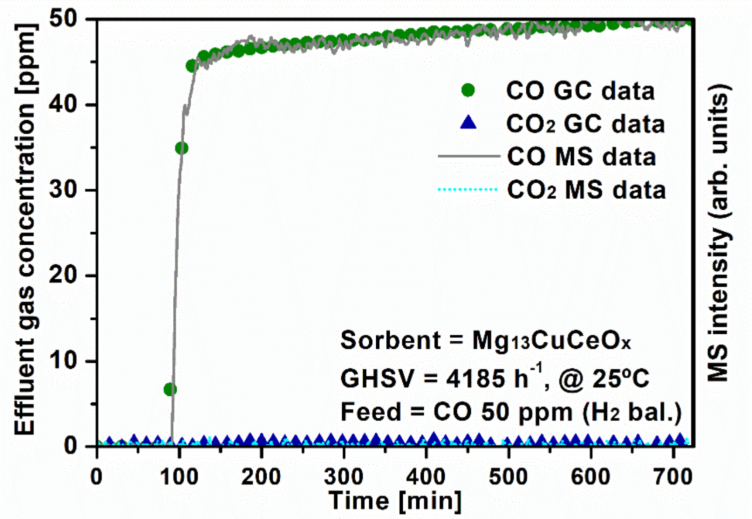


**Supplementary Figure 5.** CO breakthrough curves at 25°C under a flow of 50 ppm CO and $H_2$ balance (GHSV = 4185 h−1).

The breakthrough test was conducted under a harsher flow condition than the long-term breakthrough experiment displayed in Fig. 3d. The analysis system showed a noise level signal from the feed gas because of the $CO_2$ impurity in the feed gas. Then, through the breakthrough experiment, the noise level $CO_2$ signal did not change. The results revealed no detection of $CO_2$ even at $CO_2$ at ppb levels, and the breakthrough curve could reach 50 ppm of CO (the feed concentration). Considering the analysis system, which could detect $CO_2$ at ppb level, the MgCuCeO$_x$ sorbent satisfied the removal of trace CO in $H_2$ for a fuel-cell grade $H_2$ production (a PEMFC, i.e., 0.2 ppm of CO and 2 ppm of $CO_2$ in ISO 14687:2019)

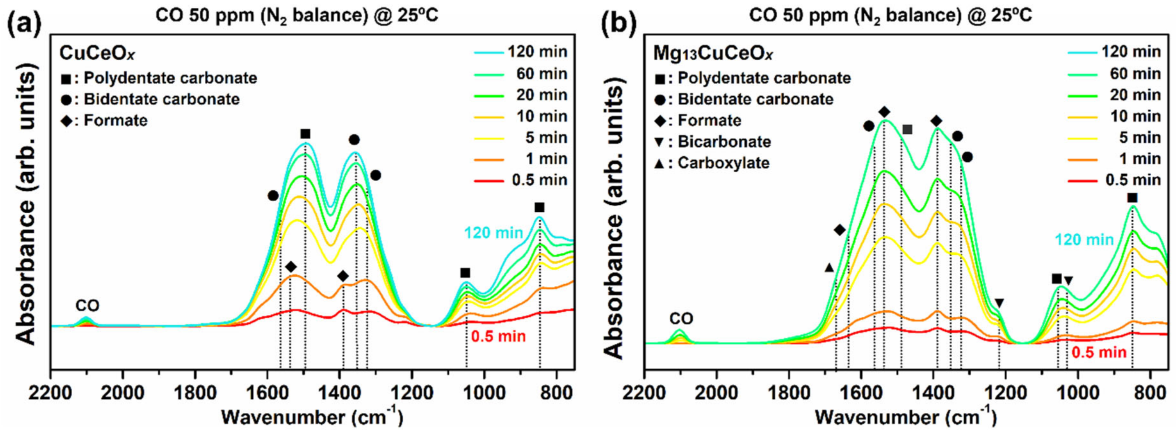


**Supplementary Figure 6.** *In-situ* DRIFTS spectra for (a) $CuCeO_x$ and (b) $Mg_{13}CuCeO_x$ at 25°C in 50 ppm CO ($N_2$ balance).

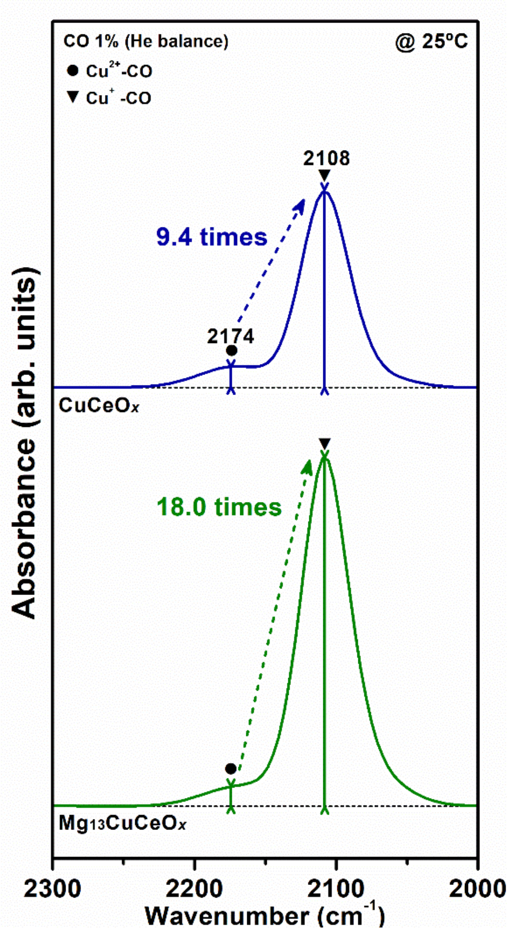


**Supplementary Figure 7**. An zoomed-in view of Fig. 6a,b: DRIFTS spectra of CO sorbed on $CuCeO_x$ and $Mg_{13}CuCeO_x$

**Supplementary Table 1.** $H_2$ purity and CO concentration in effluent gas from $H_2$ production and purification processes

| Process | Feed | $H_2$ purity [%] | CO concentration in produced $H_2$ | | Supplementary References |
|---|---|---|---|---|---|
| SMR + WGS | NG | 75–80 | 0.1–4 | % | [7–10] |
| PSA | SMR syngas | 99.959 | - | | [10] |
| PSA | SMR syngas | 99.884–99.987 | 0.78–27.05 | ppm | [11] |
| PSA | Coal off-gas | 99.96–99.99 | 1.1–6.7 | ppm | [12] |
| VSA | SMR syngas | 99.933–99.991 | 0.18–15.55 | ppm | [11] |
| TSA | SMR syngas | 99.903–99.992 | 0.17–33.42 | ppm | [11] |
| VPSA | SMR off-gas | 99.981 | 63 | ppm | [13] |

**Supplementary Table 2.** Infrared band assignments for CO chemisorption on $CuCeO_x$ and $MgCuCeO_x$.

| Surface species | Schematic structure | Peaks ($cm^{-1}$) | Symbol | Supplementary References |
|---|---|---|---|---|
| Polydentate carbonate | 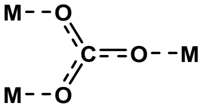 | 856<br>1053<br>1488 | ■ | [14–16]<br>[16–18]<br>[17,19–23] |
| Bidentate carbonate | 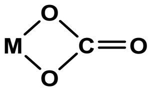 | 1323~1350<br>1563 | ● | [23–27]<br>[26] |
| Formate | 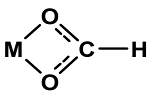 or 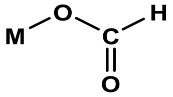 | 1392<br>1535<br>1635 | ◆ | [24,28,29]<br>[16,21,30]<br>[31] |
| Bicarbonate | 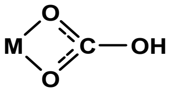 or 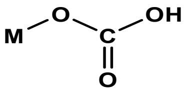 | 1038<br>1218 | ▼ | [16]<br>[29,32–34] |
| Carboxylate | 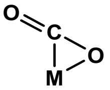 | 1670 | ▲ | [26,33,35–37] |

## Supplementary references


1. Nguyen, X. C., Kang, J.-H., Bang, G., Kim, K.-M. & Lee, C.-H. Pelletized activated carbon-based CO-selective adsorbent with highly oxidation-stable and aggregation-resistant Cu(I) sites. *Chem. Eng. J.* **451**, 138758 (2023).
2. Trogadas, P., Parrondo, J. & Ramani, V. $CeO_2$ surface oxygen vacancy concentration governs in situ free radical scavenging efficacy in polymer electrolytes. *ACS Appl. Mater. Interfaces* **4**, 5098–5102 (2012).
3. Chen, X. *et al.* The effect of the $Cu^{+}/Cu^{2+}$ ratio on the redox reactions by nanoflower CuNiOS catalysts. *Chem. Eng. Sci.* **194**, 105–115 (2019).
4. Jin, S., Byun, H. & Lee, C.-H. Enhanced oxygen mobility of nonreducible MgO-supported Cu catalyst by defect engineering for improving the water-gas shift reaction. *J. Catal.* **400**, 195–211 (2021).
5. Liu, Y.-J. *et al.* Sustained release catalysis: Dynamic copper releasing from stoichiometric spinel $CuAl_2O_4$ during methanol steam reforming. *Appl. Catal. B Environ.* **323**, 122043 (2023).
6. Ko, K.-J. *et al.* Overview of carbon monoxide adsorption performance of pristine and modified adsorbents. *J. Chem. Eng. Data* **67**, 1599–1616 (2022).
7. Papadias, D. D., Ahmed, S., Kumar, R. & Joseck, F. Hydrogen quality for fuel cell vehicles – A modeling study of the sensitivity of impurity content in hydrogen to the process variables in the SMR–PSA pathway. *Int. J. Hydrog. Energy* **34**, 6021–6035 (2009).
8. Oh, H.-T. *et al.* Pre-combustion $CO_2$ capture using amine-based absorption process for blue $H_2$ production from steam methane reformer. *Energy Convers. Manag.* **262**, 115632 (2022).
9. Shi, W. *et al.* Two-stage PSA/VSA to produce $H_2$ with $CO_2$ capture via steam methane

reforming (SMR). *Int. J. Hydrog. Energy* **43**, 19057–19074 (2018).

10. Li, H. *et al.* Modelling and simulation of two-bed PSA process for separating $H_2$ from methane steam reforming. *Chin. J. Chem. Eng.* **27**, 1870–1878 (2019).
11. Golmakani, A., Fatemi, S. & Tamnanloo, J. Investigating PSA, VSA, and TSA methods in SMR unit of refineries for hydrogen production with fuel cell specification. *Sep. Purif. Technol.* **176**, 73–91 (2017).
12. You, Y.-W. *et al.* $H_2$ PSA purifier for CO removal from hydrogen mixtures. *Int. J. Hydrog. Energy* **37**, 18175–18186 (2012).
13. Lopes, F. V. S., Grande, C. A. & Rodrigues, A. E. Activated carbon for hydrogen purification by pressure swing adsorption: Multicomponent breakthrough curves and PSA performance. *Chem. Eng. Sci.* **66**, 303–317 (2011).
14. Lin, L. *et al.* In Situ Characterization of Cu/$CeO_2$ Nanocatalysts for $CO_2$ Hydrogenation: Morphological Effects of Nanostructured Ceria on the Catalytic Activity. *J. Phys. Chem. C* **122**, 12934–12943 (2018).
15. Meunier, F. C., Tibiletti, D., Goguet, A., Reid, D. & Burch, R. On the reactivity of carbonate species on a Pt/$CeO_2$ catalyst under various reaction atmospheres: Application of the isotopic exchange technique. *Appl. Catal. Gen.* **289**, 104–112 (2005).
16. Jacobs, G., Keogh, R. A. & Davis, B. H. Steam reforming of ethanol over Pt/ceria with co-fed hydrogen. *J. Catal.* **245**, 326–337 (2007).
17. Luo, J.-Y., Meng, M., Zha, Y.-Q. & Guo, L.-H. Identification of the active sites for CO and $C_3H_8$ total oxidation over nanostructured $CuO-CeO_2$ and $Co_3O_4-CeO_2$ catalysts. *J. Phys. Chem. C* **112**, 8694–8701 (2008).
18. Binet, C., Daturi, M. & Lavalley, J.-C. IR study of polycrystalline ceria properties in oxidised and reduced states. *Catal. Today* **50**, 207–225 (1999).

19. Golovanova, V. *et al.* Effects of solar irradiation on thermally driven $CO_2$ methanation using Ni/$CeO_2$–based catalyst. *Appl. Catal. B Environ.* **291**, 120038 (2021).

20. Sheng, Z., Kim, H.-H., Yao, S. & Nozaki, T. Plasma-chemical promotion of catalysis for $CH_4$ dry reforming: unveiling plasma-enabled reaction mechanisms. *Phys. Chem. Chem. Phys.* **22**, 19349–19358 (2020).

21. Cao, T. *et al.* An in situ DRIFTS mechanistic study of $CeO_2$-catalyzed acetylene semihydrogenation reaction. *Phys. Chem. Chem. Phys.* **20**, 9659–9670 (2018).

22. Chen, S., Luo, L., Jiang, Z. & Huang, W. Size-dependent reaction pathways of low-temperature CO oxidation on Au/$CeO_2$ catalysts. *ACS Catal.* **5**, 1653–1662 (2015).

23. Gamarra, D. & Martínez-Arias, A. Preferential oxidation of CO in rich $H_2$ over CuO/$CeO_2$: Operando-DRIFTS analysis of deactivating effect of $CO_2$ and $H_2O$. *J. Catal.* **263**, 189–195 (2009).

24. Sun, S. *et al.* Low-temperature CO oxidation on CuO/$CeO_2$ catalysts: the significant effect of copper precursor and calcination temperature. *Catal. Sci. Technol.* **5**, 3166–3181 (2015).

25. Sato, A. G., Volanti, D. P., de Freitas, I. C., Longo, E. & Bueno, J. M. C. Site-selective ethanol conversion over supported copper catalysts. *Catal. Commun.* **26**, 122–126 (2012).

26. Yang, C.-C., Yu, Y.-H., van der Linden, B., Wu, J. C. S. & Mul, G. Artificial photosynthesis over crystalline $TiO_2$-based catalysts: Fact or fiction? *J. Am. Chem. Soc.* **132**, 8398–8406 (2010).

27. Bailey, S., Froment, G. F., Snoeck, J. W. & Waugh, K. C. A DRIFTS study of the morphology and surface adsorbate composition of an operating methanol synthesis catalyst. *Catal. Lett.* **30**, 99–111 (1994).

28. Wang, X., Shi, H., Kwak, J. H. & Szanyi, J. Mechanism of $CO_2$ hydrogenation on Pd/$Al_2O_3$ catalysts: Kinetics and transient DRIFTS-MS studies. *ACS Catal.* **5**, 6337–6349 (2015).

29. Bera, P., Cámara, A. L., Hornés, A. & Martínez-Arias, A. Comparative in situ DRIFTS-MS study of $^{12}CO$- and $^{13}CO$-TPR on $CuO/CeO_2$ catalyst. *J. Phys. Chem. C* **113**, 10689–10695 (2009).

30. Proaño, L. *et al.* In-situ DRIFTS study of two-step $CO_2$ capture and catalytic methanation over Ru,"$Na_2O$"/$Al_2O_3$ Dual Functional Material. *Appl. Surf. Sci.* **479**, 25–30 (2019).

31. Chen, B., Zhu, X., Crocker, M., Wang, Y. & Shi, C. Complete oxidation of formaldehyde at ambient temperature over $\gamma$-$Al_2O_3$ supported Au catalyst. *Catal. Commun.* **42**, 93–97 (2013).

32. Zhang, Z. *et al.* Support-dependent rate-determining step of $CO_2$ hydrogenation to formic acid on metal oxide supported Pd catalysts. *J. Catal.* **376**, 57–67 (2019).

33. Liu, L., Zhao, C. & Li, Y. Spontaneous dissociation of $CO_2$ to CO on defective surface of Cu(I)/$TiO_{2-x}$ nanoparticles at room temperature. *J. Phys. Chem. C* **116**, 7904–7912 (2012).

34. Yoshida, Y., Arai, Y., Kado, S., Kunimori, K. & Tomishige, K. Direct synthesis of organic carbonates from the reaction of $CO_2$ with methanol and ethanol over $CeO_2$ catalysts. *Catal. Today* **115**, 95–101 (2006).

35. Ojha, N., Bajpai, A. & Kumar, S. Visible light-driven enhanced $CO_2$ reduction by water over Cu modified S-doped g-$C_3N_4$. *Catal. Sci. Technol.* **9**, 4598–4613 (2019).

36. Liu, L., Zhao, H., Andino, J. M. & Li, Y. Photocatalytic $CO_2$ reduction with $H_2O$ on $TiO_2$ nanocrystals: Comparison of anatase, rutile, and brookite polymorphs and exploration of surface chemistry. *ACS Catal.* **2**, 1817–1828 (2012).

37. Piccolo, L., Daly, H., Valcarcel, A. & Meunier, F. C. Promotional effect of $H_2$ on CO oxidation over Au/$TiO_2$ studied by operando infrared spectroscopy. *Appl. Catal. B Environ.* **86**, 190–195 (2009).
